# First Report of the Colistin Resistance Gene *mcr-10.1* Carried by Inc_pA1763-KPC_ Plasmid pSL12517-mcr10.1 in Enterobacter cloacae in Sierra Leone

**DOI:** 10.1128/spectrum.01127-22

**Published:** 2022-06-13

**Authors:** Jiayao Guan, Letian Li, Lin Zheng, Gejin Lu, Ying Wang, Sulaiman Lakoh, Stephen Sevalie, Bowen Jiang, Xue Ji, Yang Sun, Jun Liu, Lingwei Zhu, Xuejun Guo

**Affiliations:** a Key Laboratory of Jilin Province for Zoonosis Prevention and Control, Changchun Veterinary Research Institute, Chinese Academy of Agricultural Sciences, Changchun, Jilin, China; b College of Medicine and Allied Health Sciences, University of Sierra Leone, Freetown, Sierra Leone; c Ministry of Health and Sanitation, Government of Sierra Leone, Freetown, Sierra Leone; University of Pittsburgh School of Medicine

**Keywords:** *Enterobacter cloacae*, colistin resistance, *mcr-10.1*, Inc_pA1763-KPC_ plasmid

## Abstract

Mobile colistin resistance (*mcr*) gene *mcr-10.1* has been distributed widely since it was initially identified in 2020. The aim of this study was to report the first *mcr-10.1* in Africa and the first *mcr* in Sierra Leone; furthermore, we presented diverse modular structures of *mcr-10.1* loci. Here, the complete sequence of one *mcr-10.1*-carrying plasmid in one clinical Enterobacter cloacae isolate from Sierra Leone was determined. Detailed genetic dissection and comparison were applied to this plasmid, together with a homologous plasmid carrying *mcr-10.1* from GenBank. Moreover, a genetic comparison of 19 *mcr-10.1* loci was performed. In this study, *mcr-10.1* was carried by an Inc_pA1763-KPC_ plasmid from one Enterobacter cloacae isolate. A total of 19 *mcr-10.1* loci displayed diversification in modular structures through complex transposition and homologous recombination. A site-specific tyrosine recombinase XerC was located upstream of *mcr-10.1*, and at least one insertion sequence element was inserted adjacent to a conserved *xerC*-*mcr-10.1*-*orf336*-*orf177* region. Integration of *mcr-10.1* into a different gene context and carried by various Inc plasmids contributed to the wide distribution of *mcr-10.1* and enhanced the ability of bacteria to survive under colistin selection pressure.

**IMPORTANCE** Colistin is used as one of the last available choices of antibiotics for patients infected by carbapenem-resistant bacterial strains, but the unrestricted use of colistin aggravated the acquisition and dissemination of mobile colistin resistance (*mcr*) genes. So far, 10 *mcr* genes have been reported in four continents around the world. This study presented one *mcr-10.1*-carrying Enterobacter cloacae isolate from Sierra Leone. The *mcr-10.1* gene was identified on an Inc_pA1763-KPC_ plasmid. According to the results of genetic comparison of 19 *mcr-10.1* loci, the *mcr-10.1* gene was found to be located in a conserved *xerC*-*mcr-10.1*-*orf336*-*orf177* region, and at least one insertion sequence element was inserted adjacent to this region. To our knowledge, this is the first report of identifying the *mcr-10.1* gene in Africa and the *mcr* gene in Sierra Leone.

## INTRODUCTION

Colistin is one of the last choices of antibiotic to treat severe Gram-negative bacterial infections of humans, especially infections caused by bacteria with reduced susceptibility to carbapenem antibiotics, and it has been used in livestock for more than 60 years in most countries of the world (1). *Morganellaceae*, the Burkholderia cepacia complex, and Serratia marcescens are intrinsically resistant to colistin due to the presence of the cell wall that inhibits colistin binding with the susceptible lipid target site or the lipid A modification to reduce binding (2). Recently, the unrestricted use of colistin aggravated the acquisition and dissemination of mobile colistin resistance (*mcr*) genes in *Enterobacteriaceae* (3–5), *Moraxellaceae* (6), *Morganellaceae* (6, 7), *Aeromonas* (7), *Alcaligenes* (8), *Cupriavidus* (9), Pseudomonas (6), *Serratia* (6), *Shewanella* (6), and *Vibrio* (6). The *mcr* genes encode phosphoethanolamine (PEA) transferases that catalyze the combination of PEA with lipid A and thus modify the structure of lipid A to reduce the binding affinity to colistin (10). So far, 10 *mcr* genes, including *mcr-1* to *mcr-10* with different subvariants, have been reported in four continents around the world (11).

The *mcr-10* gene was first identified in an IncFIA plasmid, pMCR10_090065, from Enterobacter roggenkampii in China in 2020 (11). Since then, *mcr-10* has been found in IncFIB (12), IncFII:IncFIA (13), IncFII:IncFIB, and IncFIB:IncFIA plasmids from Asia, Europe, Oceania, and North America, but not from Africa, South America, and Antarctica (11).

In Africa, seven (except for *mcr-6*, *mcr-7*, and *mcr-10*) of the 10 *mcr* genes have been found in IncFIB, IncFII, IncHI, IncI, IncN, IncP, IncR, and IncX plasmids from Escherichia coli, Klebsiella pneumoniae, Pseudomonas aeruginosa, Pseudomonas putida, Pseudomonas luteola, Enterobacter hormaechei, Acinetobacter baumannii, Citrobacter werkmanii, and Alcaligenes faecalis (8, 14). These *mcr*-carrying bacteria were isolated from human, animals, plants, and contaminated soil, water, and wildlife ecosystems. So far, none of *mcr* genes have been reported in Sierra Leone (8).

This study presented the complete sequence of one *mcr-10.1*-carrying plasmid in one sequenced clinical Enterobacter cloacae isolate from Sierra Leone. Detailed genetic dissection and comparison were applied to this plasmid, together with a plasmid carrying *mcr-10.1* from GenBank. Moreover, a genetic comparison of 19 *mcr-10.1* loci was performed to present diversification in modular structures of *mcr-10.1*. To our knowledge, this is the first report of identifying the *mcr-10.1* gene in Africa and the *mcr* gene in Sierra Leone.

## RESULTS

### Identification and antimicrobial susceptibility of Enterobacter cloacae SL12517.

Strain SL12517 has a 98.74% average nucleotide identity (ANI) value with the reference strain Enterobacter cloacae ATCC 13047 (accession number CP001918). Multilocus sequencing typing (MLST) analysis revealed that strain SL12517 belonged to sequence type 850 (ST850).

Strain SL12517 was resistant to colistin (MIC, 8 μg/mL), cefazolin (MIC, ≥64 μg/mL), gentamicin (MIC, ≥16 μg/mL), and trimethoprim/sulfamethoxazole (MIC, ≥320 μg/mL), intermediate to piperacillin (MIC, 32 μg/mL), tobramycin (MIC, 8 μg/mL), and nitrofurantoin (MIC, 64 μg/mL), and susceptible to piperacillin/tazobactam (MIC, ≤4 μg/mL), cefuroxime (MIC, 4 μg/mL), ceftazidime (MIC, ≤1 μg/mL), ceftriaxone (MIC, ≤1 μg/mL), cefepime (MIC, ≤1 μg/mL), aztreonam (MIC, ≤1 μg/mL), imipenem (MIC, ≤1 μg/mL), meropenem (MIC, ≤0.25 μg/mL), amikacin (MIC, ≤2 μg/mL), ciprofloxacin (MIC, ≤0.5 μg/mL), and levofloxacin (MIC, ≤1 μg/mL).

### Identification of resistance genes carried by strain SL12517.

Resistance genes carried by strain SL12517 were identified using the Comprehensive Antibiotic Resistance Database (CARD) and the ResFinder database. The chromosome of strain SL12517 carried the *bla*_CMH-3_ gene. An IncFII plasmid, pSL12517-TEM, carried *bla*_TEM-1B_ and *aacC2e* genes. An Inc_pA1763-KPC_ plasmid, pSL12517-mcr10.1, contained *mcr-10.1*, *aac2d*, *strA*, *strB*, *tetA*(D), *qnrS1*, *catA2*, *dfrA14b*, *tmrB*, and *sul2* genes. A ColRNAI plasmid, pSL12517-NR, harbored no resistance genes.

### Sequence comparison of two Inc_pA1763-KPC_ plasmids.

**FIG 1:**
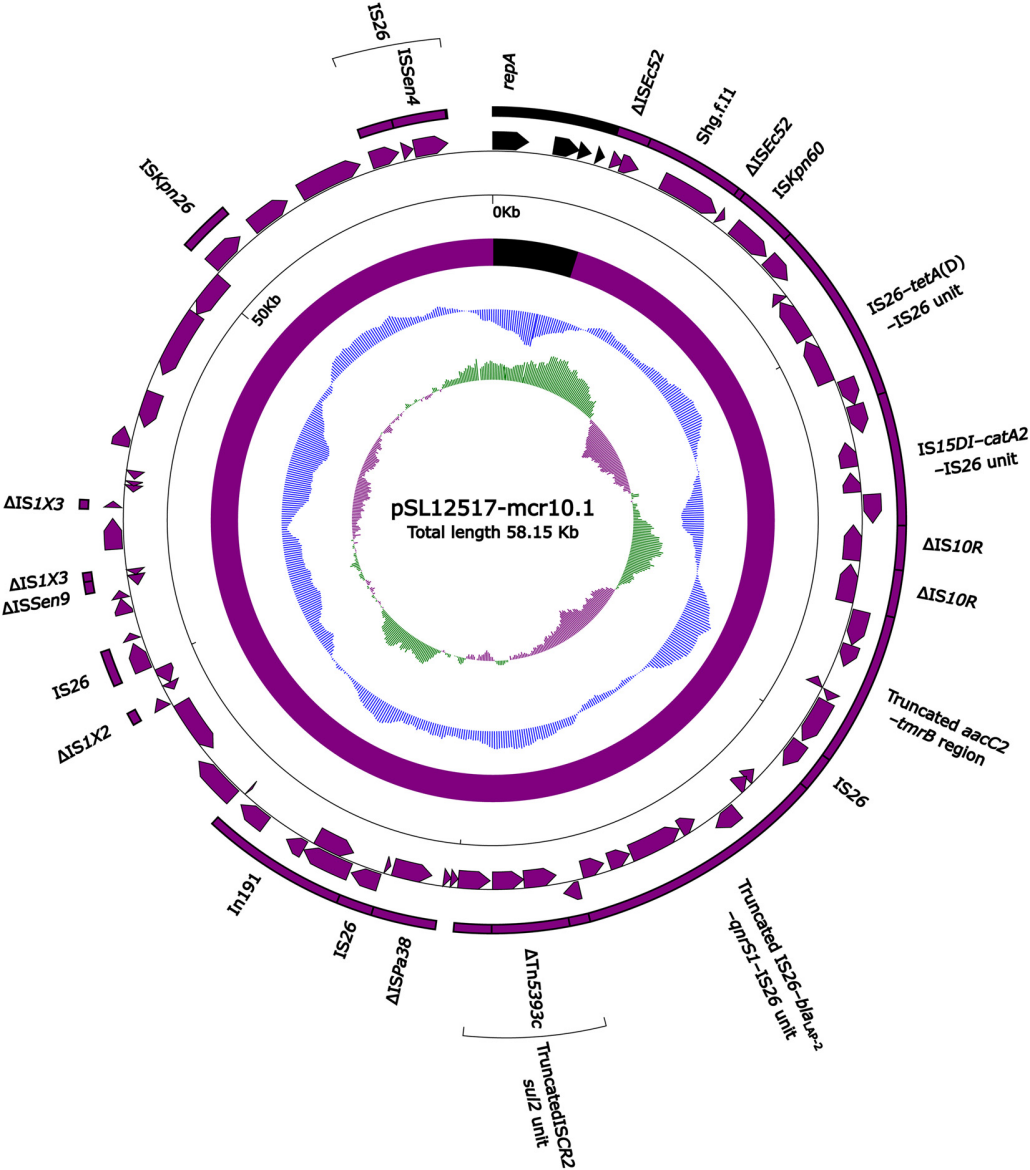
Schematic map of plasmid pSL12517-mcr10.1. Genes are denoted by arrows, and the backbone and accessory module regions are highlighted in black and purple, respectively. The innermost circle presents GC-skew [(G–C)/(G+C)], with a window size of 500 bp and a step size of 20 bp. The next-to-innermost circle presents GC content.

**FIG 2:**
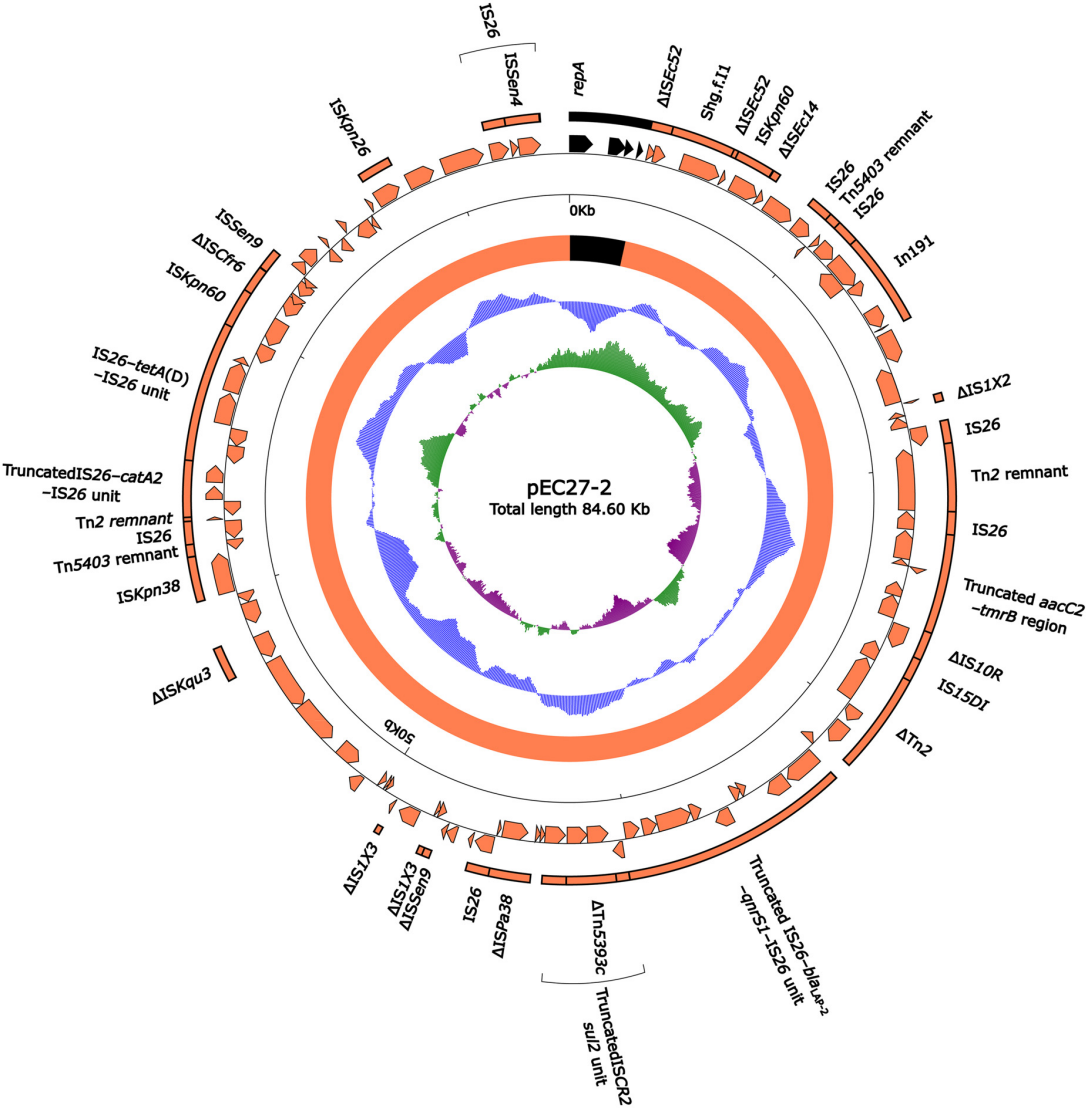
Schematic map of plasmid pEC27-2. Genes are denoted by arrows, and the backbone and accessory module regions are highlighted in black and orange, respectively. The innermost circle presents GC-skew [(G–C)/(G+C)], with a window size of 500 bp and a step size of 20 bp. The next-to-innermost circle presents GC content.

A detailed sequence comparison was applied to two *mcr-10.1*-carrying Inc_pA1763-KPC_ plasmids; one was plasmid pSL12517-mcr10.1, which was isolated from strain SL12517, sequenced here, and the other one was pEC27-2 (15) from GenBank, which was recovered from one Enterobacter cloacae isolate in Vietnam in 2010. The plasmid pSL12517-mcr10.1 shared 99.94% nucleotide identity with pEC27-2 with 99% coverage. A total of 57 and 70 open reading frames (ORFs) were predicted in pSL12517-mcr10.1 (58.1 kb long; Fig. 1) and pEC27-2 (84.6 kb long; Fig. 2), respectively. At least 12 antimicrobial resistance genes, *mcr-10.1*, *bla*_TEM-1_, *bla*_LAP-2_, *aac2d*, *strA*, *strB*, *tetA*(D), *qnrS1*, *catA2*, *dfrA14b*, *tmrB*, and *sul2*, involved in resistance to 9 different categories of antimicrobials (colistin, β-lactams, aminoglycosides, tetracycline, quinolone, chloramphenicol, trimethoprim, tunicamycin, and sulfonamide), were identified in these two plasmids.

**FIG 3:**
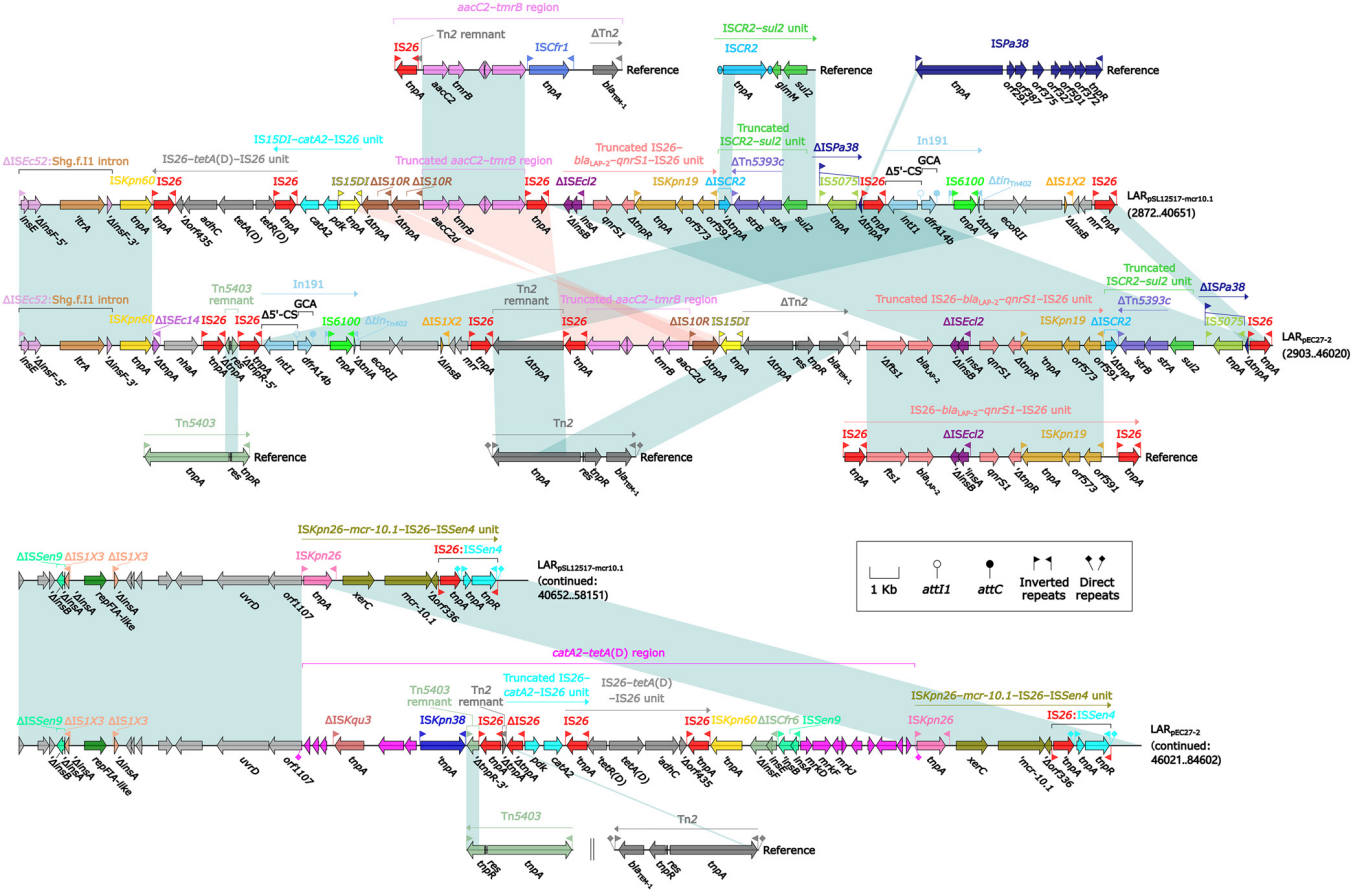
Comparison of MDR regions from pSL12517-mcr10.1 and pEC27-2. Genes are denoted by arrows. Genes, accessory genetic elements (AGEs), and other features are colored based on their functional classification. Shading in light blue or light pink denotes regions of homology (nucleotide identity ≥95%). Numbers in brackets indicate nucleotide positions within plasmids pSL12517-mcr10.1 and pEC27-2. Accession numbers of Tn*5403* (40), the *aacC2*-*tmrB* region (41), Tn*2* (42), IS*Pa38*, the IS*CR2*-*sul2* unit (43), and the IS*26* = *bla*_LAP-2_-*qnrS1*-IS*26* unit (40) used as reference are KJ958926, JX101693, HM749967, CP003149, AE014073, and HF545433, respectively.

The two plasmids shared a small backbone region (2.8 kb in length), including *rep*_IncpA1763-KPC_, *parA*, and two undetermined genes (hypothetical proteins). Two multidrug resistance (MDR) regions (Fig. 3) MDR_pSL1217-mcr10.1_ (55.2 kb long) and MDR_pEC27-2_ (81.6 kb long) were integrated at the same site adjacent to the *rep* within the two plasmids, respectively.

MDR_pSL1217-mcr10.1_ and MDR_pEC27-2_ shared a truncated *aacC2*-*tmrB* region, a truncated IS*26*-*bla*_LAP-2_-*qnrS1*-IS*26* unit, a truncated IS*CR2*-*sul2* unit (containing the *strAB*-carrying ΔTn*5393c*), a concise class 1 integron In191 with the gene cassette array (GCA) *dfrA14b*, and IS*Kpn26*-*mcr-10.1*-IS*26*-IS*Sen4* unit, but each of them integrated two additional resistance loci: (i) the IS*26-tetA*(D)-IS*26* unit and IS*15DI*-*catA2*-IS*26* unit in MDR_pSL1217-mcr10.1_ and (ii) the ΔTn*2* and *catA2*-*tetA*(D) region (bracketed by the same 4-bp direct repeats [DRs]; target site duplication signals for transposition) in MDR_pEC27-2_. Notably, 8 and 12 copies of IS*26*, IS*15DI*, and IS*6100* were presented in MDR_pSL1217-mcr10.1_ and MDR_pEC27-2_, respectively, all of which belonged to the IS*6* family and carried almost identical 14-bp inverted repeat (IR) sequences. It showed that these IS elements participate in complex homologous recombination events and promote the assembly of complex mosaic structures as observed in MDR_pSL1217-mcr10.1_ and MDR_pEC27-2_ (16).

### Comparison of 19 *mcr-10.1* loci from 19 plasmids.

**FIG 4:**
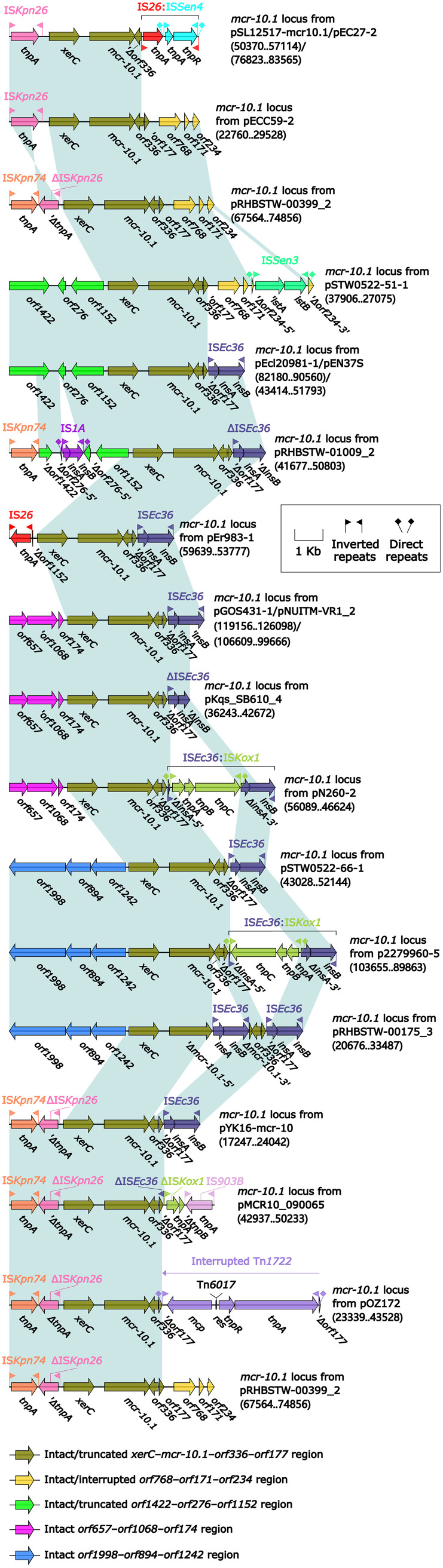
Comparison of 19 *mcr-10.1* loci from 19 plasmids. Genes are denoted by arrows. Genes, AGEs, and other features are colored based on their functional classification. Shading in light blue denotes regions of homology (nucleotide identity ≥95%). Numbers in brackets indicate nucleotide positions within the 19 plasmids.

Detailed genetic dissection and sequence comparison were applied to 19 *mcr-10.1* loci (Fig. 4) from 19 plasmids identified from GenBank as of 25 January 2022 (Table 1; see Table S1 in the supplemental material). Each *mcr-10.1* loci carried an intact or truncated version of *xerC* (site-specific tyrosine recombinase)-*mcr-10.1*-*orf336* (hypothetical protein)-*orf177* (hypothetical protein) region. Various insertion sequence (IS) elements, unit transposons, and undetermined genes were present upstream or downstream of the *xerC*-*mcr-10.1*-*orf336*-*orf177* region: (i) an intact IS*Kpn26* upstream of *xerC* in each *mcr-10.1* loci from pECC59-2, pSL12517-mcr10.1, and pEC27-2; (ii) an *orf1422*-*orf276*-*orf1152* region upstream of *xerC* in *mcr-10.1* loci from pSTW0522-51-1, pEcl20981-1, and pEN37S, and a truncated *orf1422*-*orf276*-*orf1152* region upstream of *xerC* in each *mcr-10.1* loci from pRHBSTW-01009_2 and pEr983-1 (10); (iii) an *orf657*-*orf1068*-*orf174* region upstream of *xerC* in *mcr-10.1* loci from pGOS431-1, pNUITM-VR1_2, pKqs_SB610_4, and pN260-2 (12); (iv) an *orf1998*-*orf894*-*orf1242* region upstream of *xerC* in *mcr-10.1* loci from pSTW0522-66-1, p2279960-5, and pRHBSTW-00175_3; (v) an incomplete IS*Kpn26* truncated by IS*Kpn74* upstream of *xerC* in each *mcr-10.1* loci from pRHBSTW-00399_2, pOZ172 (13), pMCR10_090065, and pYK16-mcr-10 (17); (vi) IS*26*, contributing to truncation of *orf336* in *mcr-10.1* loci from pSL12517-mcr10.1 and pEC27-2; (vii) an *orf768*-*orf171*-*orf234* region downstream of *orf177* in *mcr-10.1* loci from pECC59-2 and pRHBSTW-00399_2, and a truncated *orf768*-*orf171*-*orf234* region downstream of *orf177* in the *mcr-10.1* locus from pSTW0522-51-1; (viii) intact or truncated IS*Ec36*, leading to truncation of *orf177*, in *mcr-10.1* loci from 13 (except for pECC59-2, pRHBSTW-00399_2, pSTW0522-51-1, pOZ172, pEC27-2, and pSL12517-mcr10.1) of 19 plasmids; and (ix) an interrupted Tn*1722*, resulting in truncation of *orf177*, in the *mcr-10.1* locus from pOZ172. These results indicated that the *xerC*-*mcr-10.1*-*orf336*-*orf177* region might be the most conserved structure, and we could not determine which *mcr-10.1* locus was the earliest among these 19 plasmids.

**TABLE 1:** General features of the 19 *mcr-10.1*-carrying plasmids^*a*^

Plasmid	GenBank accession no.	Total length (bp)	Location	Host bacterium	Reference or source^*b*^
pSL12517-mcr10.1	MW048777	58,151	Sierra Leone	Enterobacter cloacae SL12517	This study
pEC27-2	CP020091	84,602	Vietnam	Enterobacter cloacae PIMB10EC27	15
pECC59-2	CP080472	64,293	China	Enterobacter hormaechei ECC59	NA
pRHBSTW-00399_2	CP056561	137,623	UK	Enterobacter cloacae RHBSTW-00399	NA
pSTW0522-51-1	AP022432	159,829	Japan	Enterobacter kobei STW0522-51	Not applicable
pEcl2098-1	CP048651	161,986	China	Enterobacter roggenkampii Ecl_20_981	NA
pEN37S	AP024497	70,277	Japan	Enterobacter cloacae En37	NA
pRHBSTW-01009_2	CP056127	70,650	UK	Enterobacter asburiae RHBSTW-01009	NA
pEr983-1	CP060738	100,102	China	Enterobacter roggenkampii Ecl-983	10
pGOS431-1	CP023893	231,294	Canada	Raoultella ornithinolytica FDAARGOS_431	NA
pNUITM-VR1_2	AP025011	261,835	Vietnam	Raoultella ornithinolytica NUITM-VR1	NA
pKqs_SB610_4	CP084774	124,980	Netherlands	Klebsiella quasipneumoniae SB610	NA
pN260-2	AP023449	244,996	Japan	Enterobacter roggenkampii OIPH-N260	12
pSTW0522-66-1	AP022466	324,199	Japan	Enterobacter roggenkampii STW0522-66	NA
p2279960-5	LR890193	120,029	Australia	K. pneumoniae INF133-sc-2279960	NA
pRHBSTW-00175_3	CP055932	68,715	UK	Enterobacter sp. strain RHBSTW-00175	NA
pYK16-mcr-10	MT468575	117,855	China	Enterobacter roggenkampii YK16	17
pMCR10_090065	CP045065	71,775	China	Enterobacter roggenkampii WCHER090065	11
pOZ172	CP016763	127,005	China	Citrobacter freundii B38	13

a All the completely sequenced and nonredundant *mcr-10.1*-carrying plasmids available in GenBank (last accessed 25 January 2022) are included. Three unnamed plasmids from strain Ecl_20_981, FDAARGOS_431, and INF133-sc-2279960 were here named pEcl20981-1, pGOS431-1, and p2279960-5, respectively.

b NA, not applicable.

### Conjugation experiments.

We failed to obtain transconjugants containing *mcr-10.1* no matter how many times the conjugation experiments were performed, which might be because the essential conjugal transfer genes, including *rlx* (relaxase), *oriT* (origin of conjugative replication), *pri* (DNA primase), *cpl* (coupling protein), and type IV secretion system (T4SS), were absent in pSL12517-mcr10.1.

## DISCUSSION

Enterobacter cloacae is a vital nosocomial pathogen and is able to cause bacteremia and other infections in humans and animals (18). Due to the wide use of antibiotics, multidrug-resistant, especially carbapenem-resistant, Enterobacter cloacae emerged (19); therefore, colistin is used as one of the last available choices of antibiotics for patients infected by carbapenem-resistant strains (20). However, *mcr*-carrying *Enterobacteriaceae* have been identified all over the world recently (9, 21, 22). This study presented the complete sequence of one *mcr-10.1*-carrying Inc_pA1763-KPC_ plasmid in one sequenced Enterobacter cloacae isolate from Sierra Leone. Detailed genetic dissection and comparison were applied to this plasmid, together with a homologous Inc_pA1763-KPC_ plasmid carrying *mcr-10.1* from GenBank. Moreover, a genetic comparison of 19 *mcr-10.1* loci was performed to display diversification in modular structures of *mcr-10.1*.

The *mcr-10.1* usually mediated low-level colistin resistance in early reports (10, 11), but strain SL12517 in this study displayed high-level colistin resistance with a MIC of 8 μg/mL. A previous study demonstrated that *mcr-10.1* was able to cofunction with *phoP* (two-component system response regulator) and *phoQ* (two-component system sensor histidine kinase) to mediate the high-level colistin resistance (10). In this study, *phoPQ* was identified on the chromosome of strain SL12517, indicating that *phoPQ* might very likely participate in the high-level colistin resistance.

The Inc_pA1763-KPC_ plasmid carried an Inc_pA1763-KPC_ replicon, which was composed of *repA_IncpA1763-KPC_* and its iterons (23). The Inc_pA1763-KPC_ replicon (previously called RepB_Rep_3-family_) was initially found in pK245 from K. pneumoniae in 2006 in Taiwan (24); since then, it has been frequently found in different plasmids in many K. pneumoniae isolates. In this study, two Inc_pA1763-KPC_ plasmids, pSL12517-mcr10.1 and pEC27-2 (15), were identified in Enterobacter cloacae recovered from Sierra Leone in 2018 and from Vietnam in 2010, respectively. Only two *mcr-10.1*-carrying Inc_pA1763-KPC_ plasmids (pSL12517-mcr10.1 and pEC27-2) have been identified until now, and no *mcr-10.1*-carrying Inc_pA1763-KPC_ plasmids were found in other species of bacteria. This result indicated that transfer of the Inc_pA1763-KPC_ plasmids without *mcr-10.1* from K. pneumoniae to Enterobacter cloacae was prior to acquisition of *mcr-10.1* by the Inc_pA1763-KPC_ plasmids. pEC27-2 was found earlier than pSL12517-mcr10.1, and colistin has not been used clinically in Sierra Leone (25); therefore, we speculate that pEC27-2 was possibly transferred from Vietnam to Sierra Leone through international food (animal- and plant-based) trade or travel (8).

According to detailed genetic dissection and comparison of 19 *mcr-10.1* loci, the genetic organization *xerC*-*mcr-10.1*-*orf336*-*orf177* might be the original modular structure of the *mcr-10.1* locus. Various IS elements or transposons were inserted upstream or downstream of the *xerC*-*mcr-10.1*-*orf336*-*orf177* region, which resulted in the truncation of *orf177*, but no truncation of *xerC* was found. Some mobile genetic elements (MGEs) integrated into the chromosomes using *xerC*-encoding tyrosine recombinases in Enterobacter cloacae (26, 27). This indicated that *xerC* could participate in mobilization of *mcr-10.1* (10, 11). Diverse IS elements or transposons inserted upstream or downstream of the *xerC*-*mcr-10.1*-*orf336*-*orf177* region suggest that the area surrounding this conserved region is the high-frequency region for insertion of MGEs (3).

In conclusion, this is the first report of identifying the *mcr-10.1* gene in Africa and the *mcr* gene in Sierra Leone. The *mcr-10.1* gene was able to rely on plasmids to accomplish intercellular transfer and on site-specific tyrosine recombinase to achieve intracellular transfer. Although *mcr-10.1* was first identified in 2020, it showed the tendency of rapid propagation throughout the world due to uncontrolled colistin consumption. So far, *mcr-10.1*, which could be carried by Enterobacter cloacae, Enterobacter kobei, Enterobacter roggenkampii, Enterobacter asburiae, K. pneumoniae, Klebsiella quasipneumoniae, Raoultella ornithinolytica, and Citrobacter freundii, had been found in Sierra Leone, China, Japan, Vietnam, the United Kingdom, Netherlands, Canada, and Australia. It could be captured by various MGEs and integrated in diverse types of plasmids. Particularly, it should be noted that the high MIC value due to *mcr-10.1* might enhance the ability of bacteria to survive under colistin selection pressure and aggravate the difficulty in treating infections caused by *mcr-10.1*-carrying bacteria, especially in low-income countries. Therefore, it is necessary to continuously monitor the spread of *mcr-10.1* in the future.

## MATERIALS AND METHODS

### Bacterial isolation and identification.

Strain SL12517 was recovered from a public hospital in Sierra Leone in 2018 (28). The MIC of colistin was determined by the broth microdilution method according to Clinical and Laboratory Standards Institute (CLSI) guidelines (29). The breakpoint of colistin was defined by the European Committee on Antimicrobial Susceptibility Testing (EUCAST) (http://www.eucast.org). The Escherichia coli ATCC 25922 strain was used as a control. MICs of piperacillin, piperacillin/tazobactam, cefazolin, cefuroxime, ceftazidime, ceftriaxone, cefepime, aztreonam, imipenem, meropenem, amikacin, gentamicin, tobramycin, ciprofloxacin, levofloxacin, nitrofurantoin, and trimethoprim/sulfamethoxazole were tested using Vitek 2 and interpreted according to the CLSI guidelines (29).

### Sequencing and sequence assembly.

Bacterial genomic DNA was isolated from strain SL12517 using the UltraClean microbial kit (Qiagen, North Rhine-Westphalia, Germany), and sequenced with a PacBio RS II sequencer (Pacific Biosciences, CA, USA). The reads were assembled *de novo* utilizing SMARTdenovo (http://github.com/ruanjue/smartdenovo).

### Bacterial precise species identification and genotyping.

Bacterial precise species identification was performed using pairwise ANI analysis between strain SL12517 and the reference genome (http://www.ezbiocloud.net/tools/ani). A ≥95% ANI cutoff was used to define a bacterial species (30). Genotyping of strain SL12517 was performed by MLST at the online database PubMLST (http://pubmlst.org).

### Sequence annotation and comparison.

RAST 2.0 (31) and blastp/blastn (32) searches were used to predicted ORFs. The online databases CARD (33), ResFinder (34), ISfinder (35), INTEGRALL (36), and Tn number registry (37) were used to find resistance genes and mobile elements. Pairwise sequence comparisons were carried out with blastn. Inkscape 1.0 was used to draw gene organization diagrams (http://inkscape.org/en/).

### Conjugation experiments.

Conjugation experiments were performed with strain SL12517 used as a donor and rifampin-resistant Escherichia coli EC600 as a recipient (38, 39). Donor and recipient strains (3 mL each) were cultured overnight at 37°C and mixed together. The mixed cells were harvested by centrifugation for 3 min at 1,200 × *g*, washed with 3 mL of Luria-Bertani (LB) broth and resuspended in 150 μL of LB broth. The mixture was spotted on a 1-cm^2^ hydrophilic nylon membrane filter with a 0.45-μm pore size (Millipore), which was placed on an LB agar plate and then incubated for mating at 37°C for 6 h. The cells were recovered from the filter membrane and spotted on Muller-Hinton (MH) agar (BD Biosciences) plates containing 1,500 μg/mL rifampin and 4 μg/mL colistin for selecting an *mcr-10.1*-carrying transconjugant.

### Data availability.

The complete sequence of plasmid pSL12517-mcr10.1 has been submitted to GenBank under the accession number MW048777.
