# Supplementary material for: First Report of the Colistin Resistance Gene mcr-10.1 Carried by IncpA1763-KPC Plasmid pSL12517-mcr10.1 in Enterobacter cloacae in Sierra Leone
Source: Microbiol Spectr. 2022 Jun 13;10(4):e01127-22. doi: 10.1128/spectrum.01127-22 (PMC9431528; doi:10.1128/spectrum.01127-22)
Supplement: Supplemental file 1 — Table S1. Download spectrum.01127-22-s0001.pdf, PDF file, 0.07 MB [file spectrum.01127-22-s0001.pdf]

TABLE S1. Background information of the 19 bacterial isolates analyzed in this study

Bacterium	Isolate	Country	Hospital/farm	Year	Host	Specimen	Source
Enterobacter cloacae	SL12517	Sierra Leone	Hospital A	2018	Homo sapiens	Urine	This study
Enterobacter cloacae	PIMB10EC27	Vietnam	Hospital B	2010	Homo sapiens	Urine	GenBank
Enterobacter cloacae	RHBSTW-00399	UK	Not applicable	2017	Environment	Wastewater	GenBank
Enterobacter cloacae	En37	Japan	Not applicable	2015	Canis lupus familiaris	Pus	GenBank
Enterobacter roggenkampii	Ecl_20_981	China	Not applicable	2019	Environment	Wastewater	GenBank
Enterobacter roggenkampii	Ecl-983	China	Hospital D	2019	Environment	Wastewater	GenBank
Enterobacter roggenkampii	OIPH-N260	Japan	Not applicable	2019	Homo sapiens	Not applicable	GenBank
Enterobacter roggenkampii	YK16	China	Farm A	2019	Chicken	Anal swab	GenBank
Enterobacter roggenkampii	WCHER090065	China	Hospital F	2016	Homo sapiens	Not applicable	GenBank
Enterobacter roggenkampii	STW0522-66	Japan	Not applicable	2018	Environment	Wastewater	GenBank
Raoultella ornithinolytica	FDAARGOS_431	Canada	Hospital E	2015	Homo sapiens	Rectal	GenBank
Raoultella ornithinolytica	NUITM-VR1	Vietnam	Not applicable	2021	Not applicable	Not applicable	GenBank
Enterobacter hormaechei	ECC59	China	Hospital C	2017	Homo sapiens	Broncho-alveolar lavage	GenBank
Enterobacter asburiae	RHBSTW-01009	UK	Not applicable	2017	Environment	Wastewater	GenBank

Enterobacter kobei	STW0522-51	Japan	Not applicable	2018	Environment	Wastewater	GenBank
Enterobacter	RHBSTW-00175	UK	Not applicable	2017	Environment	Freshwater	GenBank
Klebsiella pneumoniae	INF133-sc-2279960	Australia	Not applicable	2015	Not applicable	Not applicable	GenBank
K. quasipneumoniae	SB610	Netherlands	Not applicable	2000	Environment	Water	GenBank
Citrobacter freundii	B38	China	Hospital G	1998	Homo sapiens	Not applicable	GenBank
